# Leaf Anatomy and 3-D Structure Mimic to Solar Cells with light trapping and 3-D arrayed submodule for Enhanced Electricity Production

**DOI:** 10.1038/s41598-019-46748-x

**Published:** 2019-07-16

**Authors:** Min Ju Yun, Yeon Hyang Sim, Seung I. Cha, Dong Yoon Lee

**Affiliations:** 1 Energy Conversion Research Center, Electrical Materials Research Division, Korea Electrotechnology Research Institute, Changwon, South Korea; 2 Department of Electro-functionality Materials Engineering, University of Science and Technology, Changwon, South Korea

**Keywords:** Solar energy, Electrical and electronic engineering

## Abstract

Plant leaves are efficient light scavengers. We take a ‘botanical approach’ toward the creation of next-generation photovoltaic cells for urban environments. Our cells exhibit high energy conversion efficiency under indirect weak illumination. We used two features of leaves to improve dye-sensitized solar cells (DSSCs). Leaves feature a cuticle, a covering epidermis, and palisade and spongy cells. Leaves are also carefully arrayed within the plant crown. To mimic these features, we first created a light-trapping layer on top of the solar cells and microscale-patterned the photoanodes. Then we angled the three-dimensional DSSCs to create submodules. These simple mimics afforded a 50% enhancement of simulated daily electricity production. Our new design optimizes light distribution, the photoanode structure, and the DSSC array (by creating modules), greatly improving cell performance.

## Introduction

Improved photovoltaic (PV) electricity generation in urban environments demands new approaches to solar cell construction given that the installation environments and illumination conditions differ from those of rural environments where solar plants are usually constructed. Urban PV systems must produce electricity from indirect illumination due to restricted installation areas and angles^1,2^. To meet these demands, new paradigms are required; existing systems derive electricity under strong direct illumination (such as 1 sun AM1.5 conditions) over large areas. Solar cells and modules must be redesigned for applications in urban environments. To this end, we can learn from nature^3–6^, particularly from plant structure/survival mechanisms. Plants live in both shaded and sunny areas^7–9^, and urban environments tend to have more of the former. We take a ‘botanical approach’ toward the development of next-generation PV cells suitable for urban environments. Plants evolved in terms of leaf form and photosynthetic efficiency under various environmental conditions, including shaded or cloudy climates. Plants are found in many environments, exhibiting characteristics essential for survival. By exploring the leaves of plants living in shade, we improved the conversion efficiency of solar cells; we exploited how plants survive in urban-like environmental conditions. We used two features of leaves to improve dye-sensitized solar cells (DSSCs)^10^, mimicking photosynthesis^11,12^. Leaves feature a cuticle, an upper epidermis, and palisade and spongy cells^13–15^, and are arrayed in the leafy crown in a manner resembling a Fibonacci series^16–18^. First, we created a light-trapping layer on top of the solar cells and microscale-patterned the photoanodes. This changed light distribution within the cell, particularly within the photoanode, and increased energy conversion efficiency by enhancing the utilization of incident photons. In addition, we improved charge collection capacity by restricting the carrier path. Then we mimicked the leaf array structure by angling three-dimensional (3D) arrays of DSSC submodules to maximize electricity production; we controlled light intensity and path over small areas. Mimicking and applying to DSSC of leaf anatomy structure especially epidermis and palisade structure and leaf array structure by considering light distribution with vertical and oblique incident light will be first try study.

Using these simple mimics, we found that simulated, daily electricity production was enhanced by more than 55%. We describe our new design, and emphasize that this study is but the first step in adoption of a botanical approach. Detailed optimization will follow. However, we show that our approach improves PV cells required in urban environments; the approach exhibits great potential.

## Results and Discussion

**Figure 1:**
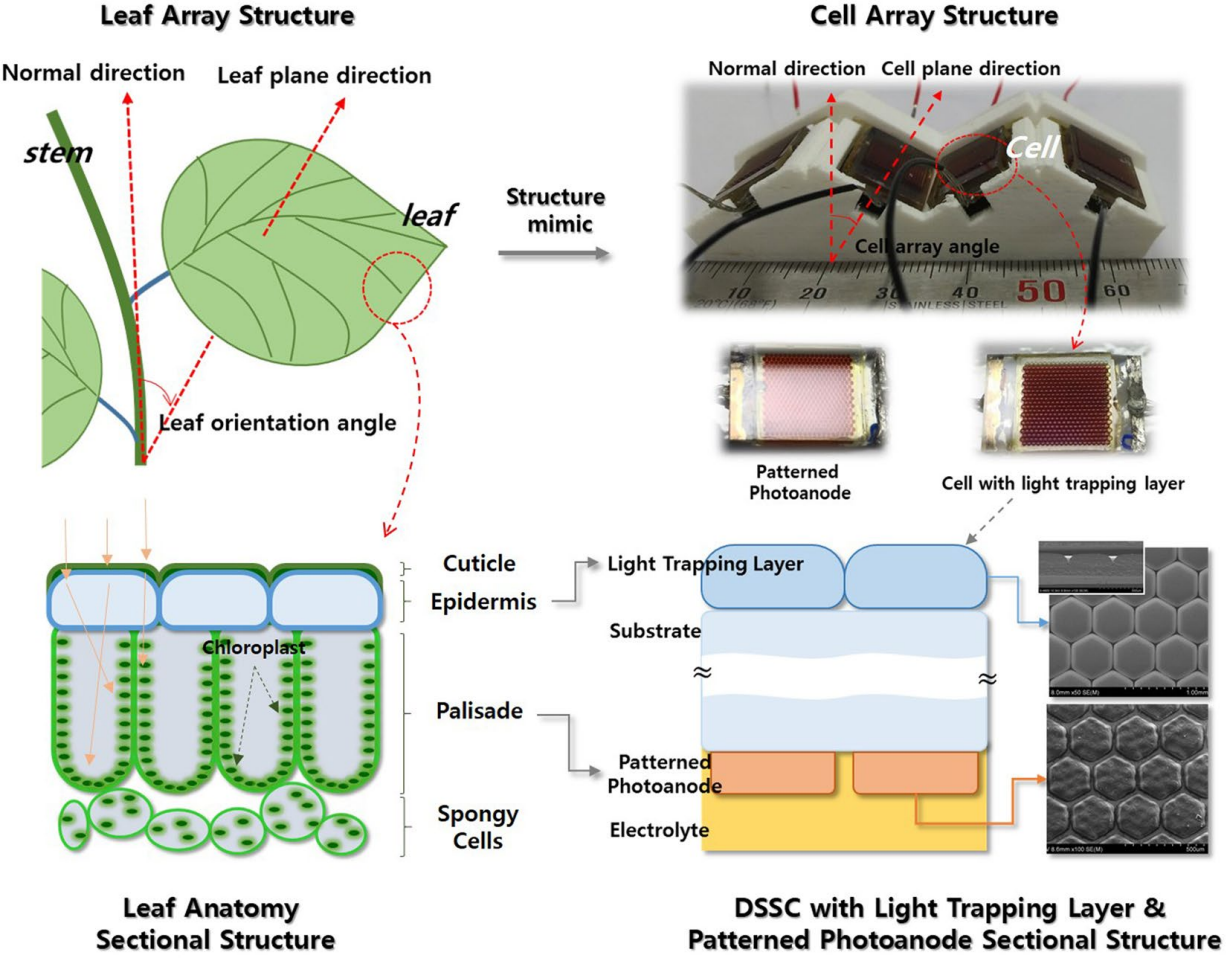
Schematic showing characteristic features of leaves and how they were applied to construct DSSCs (top left). Top right: The schematics of leaves in the plant crown and the mimicking strategies we used to create 3D arrays of DSSC submodules at various angles with and without light-trapping layers. Bottom left: Schematic of the leaf epidermis, and the palisade and sponge cells, and (bottom right) their mimics employed to create new DSSCs featuring light-trapping layers and patterned photoanodes; the detailed structures are apparent in the scanning electron micrographs.

Plants must adapt to the climate and the environment; they cannot move to escape suboptimal conditions. Leaf orientation in the crown is critical in this context. Generally, leaves are oriented at an angle to the light, not vertically (Fig. 1). This is because the crown surface area is large and angling limits the light received to that required for photosynthesis. Such an array structure is optimal for collection of scattered and indirect illuminations. In addition, as photosynthesis is a slow chain reaction, the leaf anatomy (Fig. 1) balances the number of incident photons to those consumed by photosynthesis, maximizing collection efficiency. Each leaf in the crown operates at high efficiency, using omnidirectional incident light of low intensity. We applied this structure to create 3D arrays of DSSCs that are highly efficient under weak and oblique illumination. DSSCs exhibit relatively weak absorption, and slow diffusion of electrons limits charge collection, given the extensive interfaces between titanium dioxide (TiO_2_) nanoparticles bearing the dye used for light capture, and the thickness of the photoanode used to capture photons. This situation well-matches that of leaf cells.

The 3D modular arrays of DSSCs produce higher power outputs when light is of low intensity and obliquely incident. To maximize performance, we adapted another feature of leaves: we redesigned the DSSC cell structures to resemble the leaf anatomy (Fig. 1). The leaf has a cuticle protecting tissue from strong ultraviolet light, an epidermis, palisade cells (of which most engage in photosynthesis), and spongy cells. The epidermis modifies the path of incident light so that chloroplasts on the walls of palisade cells receive homogeneously regulated light appropriate to the photosynthesis rate. The tubular shape of palisade cells aids in the distribution of light to chloroplasts. Additional photosynthesis is performed in spongy cells, whose principal function is to support leaf tissues, by providing a path for mass transportation and scattering light within the leaf. Therefore, leaf structures are optimized to capture and use omnidirectional incident light^19–22^. In addition, morphological variation in epidermal and palisade cells allows the direction and intensity of incoming light to be carefully controlled^23^.

We placed a light-trapping layer on the top of the DSSCs and microscale-patterned the photoanodes, to mimic epidermal and palisade cells, respectively (Fig. 1). The light-trapping layer mimics the leaf epidermis, modified light distribution by focusing and spreading into patterned photoanode, and patterned photoanode mimics the leaf palisade reflected and trapped incident light. At omnidirectional incident light, more trapped light enhanced solar cell performance. (Fig. S1). The patterned photoanodes mimic palisade cells, confining incoming photons and restricting the lateral movement of the generated electrons. Thus, omnidirectional light capture is maximized and recombination loss is minimized, improving energy conversion of weak, obliquely incident light, and enhancing power output.

**Figure 2:**
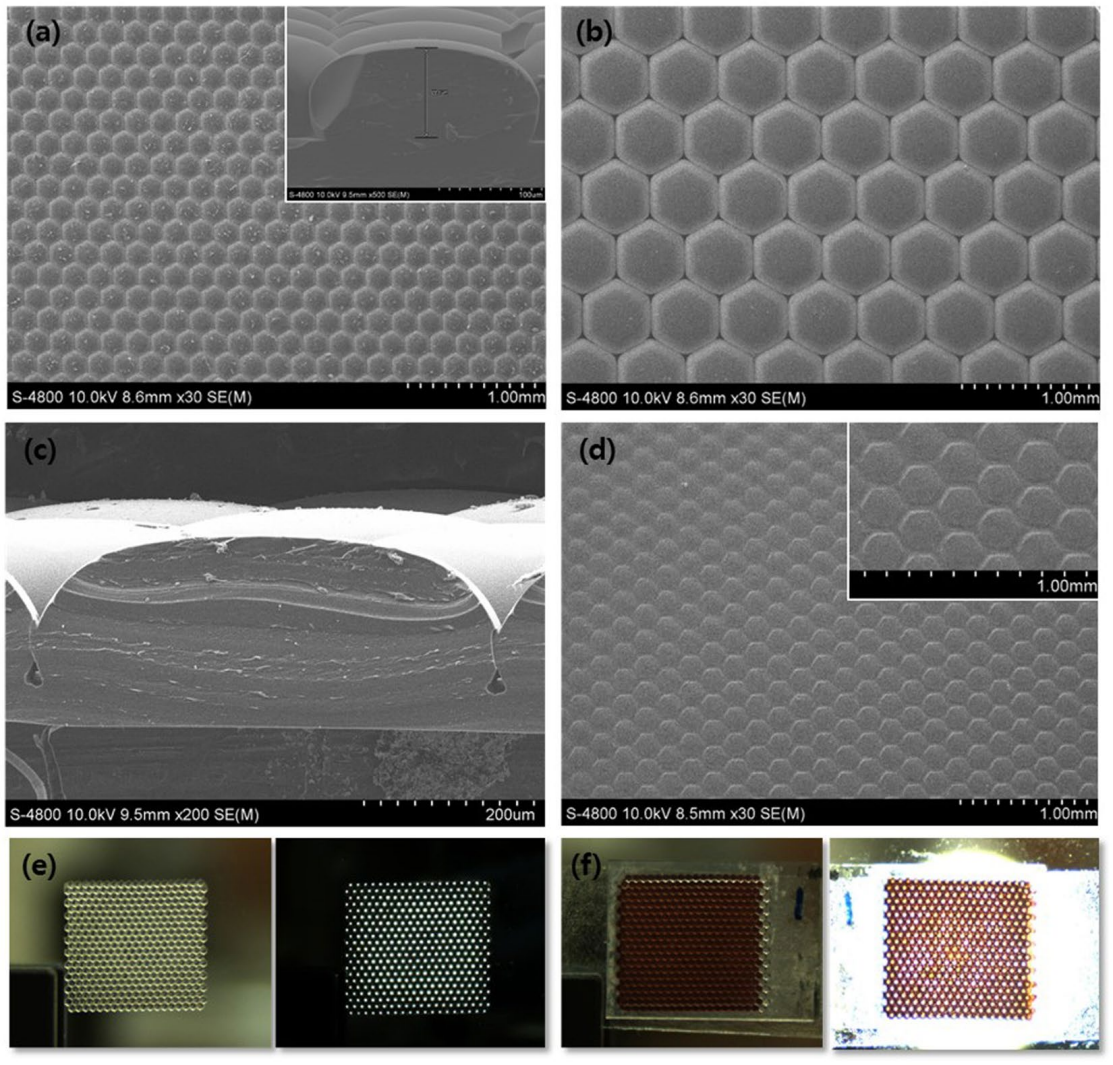
Scanning electron micrographs of the light-trapping patterns of (**a**) a 200 µm pattern (inset; cross-sectional image), (**b**) a 600 µm pattern, and (**c**) cross-sectional image. (**d**) Scanning electron micrograph of a 200 µm patterned photoanode and a magnified image (inset). Also shown are charge-coupled device (CCD) images of (**e**) the light-trapping layer of the 600 µm pattern and (**f**) the patterned photoanode in the presence of a light-trapping layer. The images on the right show the light distributions, and the focusing patterns through the light-trapping layers, derived using a light-emitting diode (LED).

The light-trapping layers were fabricated by molding etched, lens-shaped Si wafers of two diameters, 200 and 600 µm, from polydimethysiloxane (PDMS) (Fig. S2). The plane views show close-packed arrays of lenses of 200 (Fig. 2a) and 600 (Fig. 2b) µm in diameter. In the cross-sectional views, the lenses were pot-shaped (insets of Fig. 2a,c). Photoanodes were patterned via screen-printing using a mask; all patterns were clear and non-overlapping (Fig. 2d). The photoanode thickness ranged from 9 to 23 µm for the 200 µm pattern and 12 to 35 µm for the 600 µm pattern; the spacing between patterns was 30 µm. Such spacing causes photon loss and thus incoming light is not utilized. However, the light-trapping layer inhibits transmission of incoming light to the spacing regions, as shown in Fig. S3. In the 600 µm version, incident light is distributed homogeneously (Fig. 2e), scattered light is incident to the center of the layer, and all light-trapping patterns focus incident light. In the 200 µm version, the incident light is well-distributed and focused on the photoanode patterns (Fig. S4). Both light-trapping layers are designed to ensure that the pattern of trapped light is the same as that of the photoanode. Figure 2f illustrates this alignment.

We sought to optimize light-trapping and photoanode patterning by reference to light distribution, the photon travel path, and diffusion and collection of the generated electrons. However, the potential for further performance improvement is great, given the many morphologies of epidermal cells and the various geometries of palisade cells. Thus, many well-matched combinations of light-trapping/photoanode patterns may be available. However, we here focused on proof-of-concept of our botanic approach; our new DSSCs are relatively simple. We aligned the patterns linearly (Fig. 2f). Further performance improvements are eminently possible.

**Figure 3:**
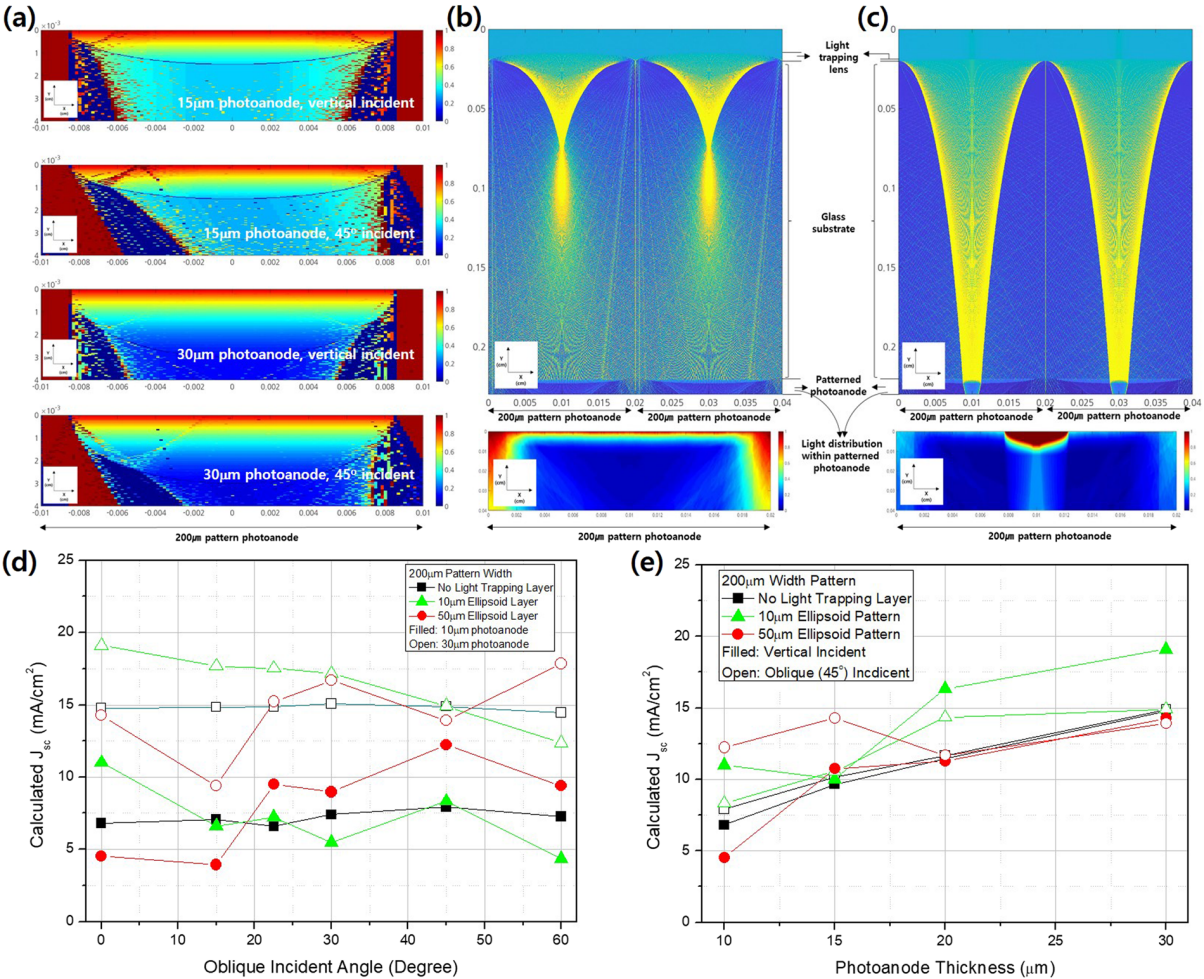
Light intensity distributions within patterned photoanodes (**a**) 15 and 30 µm in thickness in the absence of light-trapping layers for vertical and 45° obliquely incident light and (**b**,**c**) the distributions when light-trapping layers (lenses 200 µm in diameter of height 10 µm (**b**) and 50 µm (**d**) were present, as revealed by 2D ray tracing. FEM calculations showed that the current density (**d**) depended on the oblique angle of incident light when the patterned photoanodes were 10 and 30 µm in thickness and the light-trapping layers were 10 and 50 µm in thickness, and (**e**) on the thickness of the patterned photoanode when the light-trapping layers were 10 and 50 µm in thickness and light was delivered vertically or at a 45° oblique angle.

We analyzed light distributions and current densities via 2D ray tracing followed by use of a finite difference method (FEM) employing an electron continuity equation for the photoanode. This used the light distribution pattern obtained by ray tracing after introduction of the light-trapping layer and photoanode patterning. The details are described in the Experiment Details section. The light intensity distributions within 200 µm patterned photoanodes of two thicknesses, 15 and 30 µm, in the absence of light-trapping layers, are shown in Fig. 3a for vertical and 45° obliquely incident light. For both types of light, the patterned electrodes modified the light intensities and concentrated light in appropriate regions, as generally expected by the Beer-Lambert Law; the distributions were clearer and stronger for obliquely incident light. However, the points of concentrated light were located near the interfaces of the patterned photoanodes and the fluorine-doped tin oxide (FTO) glass when thinner electrodes (which more efficiently collect charge than thicker electrodes) were used. When light-trapping layers were introduced, the light distributions within patterned photoanodes exhibited considerable changes, as shown in Fig. 3b,c. When the pot-shaped light-trapping layer had a focal point within the FTO glass substrate (at a lens height of 50 µm), the incident light spread after focusing, inducing relatively homogeneous light distribution within the patterned photoanode. The light arrived at the photoanode not vertically but rather obliquely; the travel length through the patterned photoanode increased when the light was focused and distributed by the light-trapping layer. Epidermal cells play a similar role in leaves. However, when the focus was located beyond the patterned photoanode (at a lens height of 10 µm), as shown in Fig. 3c, the concentrated light passed the photoanode, and the light distribution within the photoanode differed greatly from that expected by application of the Beer-Lambert Law. Such changes in light distribution within the photoanode induced changes in photoanode current density, reflecting the balance between charge generation, recombination, and diffusion within the photoanode. To explore the effects of the incident angle, omnidirectional data were calculated as shown in Fig. 3d; the current density (Jsc) increased with increasing incident angle; the omnidirectional properties were outstanding. Here, a thin photoanode was associated with greater improvement. However, when a light-trapping layer 10 µm thick was applied, the Jsc decreased as the incident angle increased, because the focal point moved to the edge of the photoanode where the generation rate is much lower as the incident angle increases. Thus, light-trapping captured a wide range of incoming photons and spread them homogenously to where energy was generated; we enhanced performance by adopting certain aspects of leaf anatomy.

The photoanode thickness range was calculated as shown in Fig. 3e. When a light-trapping layer was absent, the Jsc increased as the photoanode thickness increased to 30 µm, thus much thicker than the non-patterned photoanode. When a light-trapping layer of 50 µm was applied, photoanodes thinner than 20 µm afforded improvements when both vertical and oblique illumination were delivered, whereas a light-trapping layer 10 µm thick was optimal when the photoanode was thicker than 20 µm. Thus, both the light-trapping layer and the patterned photoanode affected performance. The light distributions within DSSCs and the Jsc levels were sensitive to the geometries of the light-trapping layers and photoanodes, and combinations thereof. However, it is possible to control the pattern width of the light distribution layer and hence photoanode performance; further studies are necessary.

**Figure 4:**
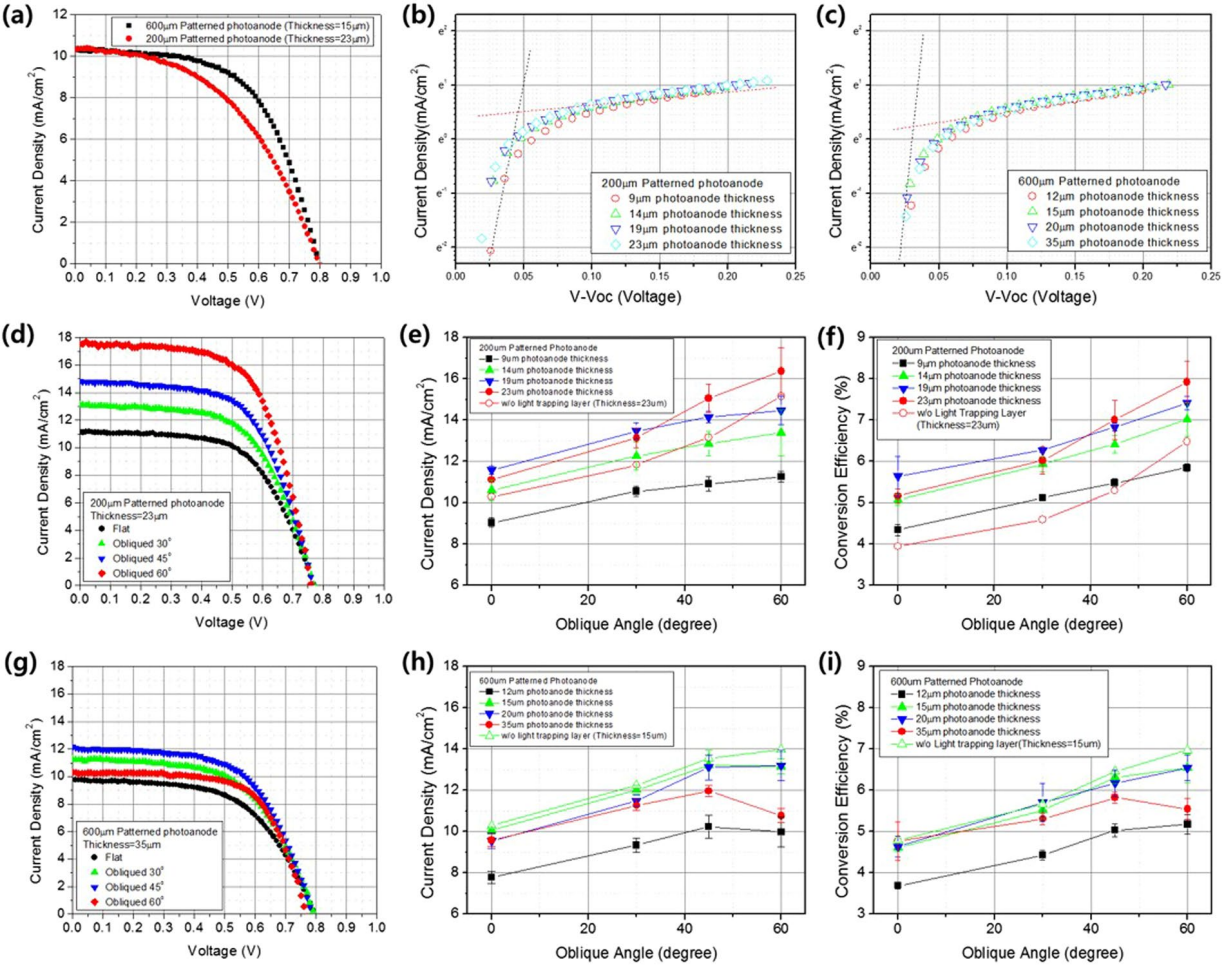
(**a**) The relationship between current density and the voltage of 200 µm patterned photoanodes (23 µm in thickness) and 600 µm patterned photoanodes (15 µm in thickness) in the absence of light-trapping layers. Also shown are the relationships between the logarithm of the current density and the differences in the open circuit voltage of (**b**) 200 µm patterned photoanodes and (**c**) 600 µm patterned photoanodes, by anode thickness. Also shown are the relationships between current density and the voltage of (**d**) 200 µm patterned photoanodes (23 µm in thickness) and (**g**) 600 µm patterned photoanodes (15 µm in thickness) angled from the vertical at 30°, 45°, and 60° oblique to the incident light. Also shown are current densities (**e**) for 200 µm patterned photoanodes and (**h**) 600 µm patterned photoanodes, and conversion efficiencies (**f**) for 200 µm patterned photoanodes and (**i**) 600 µm patterned photoanodes by photoanode thickness with light delivered vertical or at oblique angles of 30°, 45°, and 60°. All measurements were made under the 1 sun AM1.5 condition.

Previously, we reported that patterned electrodes enhanced electron diffusivity by restricting electron movement and modifying light distribution within photoanodes^24^. We prepared our patterned photoanodes by reference to these data; we used printed semi-solid electrolytes as indicated in Fig. S5. As shown in Fig. 4a, we obtained a 4% conversion efficiency using a 200 µm patterned photoanode 23 µm in thickness and a 4.8% conversion efficiency employing a 600 µm patterned photoanode 15 µm in thickness under 1 sun AM1.5 illumination conditions. Given that empty spaces exist between patterns, the use of a semi-solid electrolyte and omission of a scattering layer should improve DSSC performance. When a semi-solid electrolyte was used, the 600 µm patterned photoanode exhibited a higher recombination rate than the 200 µm patterned photoanode, and the effect of thickness was minimal. However, increasing thickness induced slightly more recombination (Fig. 4b,c).

Ray tracing (Fig. 3) showed that a light-trapping layer 50 µm in height afforded optimal omnidirectional light capturing; we thus used such a layer. We changed the patterning (width) of the photoanode to similar effect. When the pattern was 200 µm in width, an increasing incident angle increased the Jsc value (Fig. 4d,e; these values were calculated by reference to the photoanode projection area and the incident beam. The increase in Jsc as the incident angle increased did not severely degrade the fill factor or the Voc (Fig. 4d). The energy conversion efficiency shown in Fig. 4f mirrored that tendency; introduction of a light-trapping layer greatly improved efficiency. Mimicking of a leaf structure modified the Jsc by changing the light distribution and also affected DSSC operation by regulating the reaction rate. However, further study is required. In addition, as expected, during FEM simulation, a photoanode 19 µm in thickness was associated with a higher Jsc until the incident angle fell below 45°, at which time a 23 µm thick photoanode afforded better performance. Ray tracing combined with FEM analyses well-matched the experimental results and should be used for future optimization.

When the patterning was 600 µm in width, DSSC performance increased with increasing angle of oblique incident light as shown in Fig. 4g–i. The Jsc (Fig. 4h) and energy conversion efficiency (Fig. 4i) indicated that the light-trapping layer afforded lower improvements than those afforded by the 200 µm pattern. As the light distribution behavior within the 600 µm patterned photoanode indicated (Fig. S6), when a 50 µm thick light-trapping layer was present, the focused light was not spread homogeneously within the photoanode, being rather focused at the center. Thus, both recombination and a larger width had limited effects on light distribution. However, these results are greatly affected by even minute differences between the light-trapping and photoanode patterns; a strict 1:1 geometric match is required. Future research should identify other matching conditions and explore their effects.

**Figure 5:**
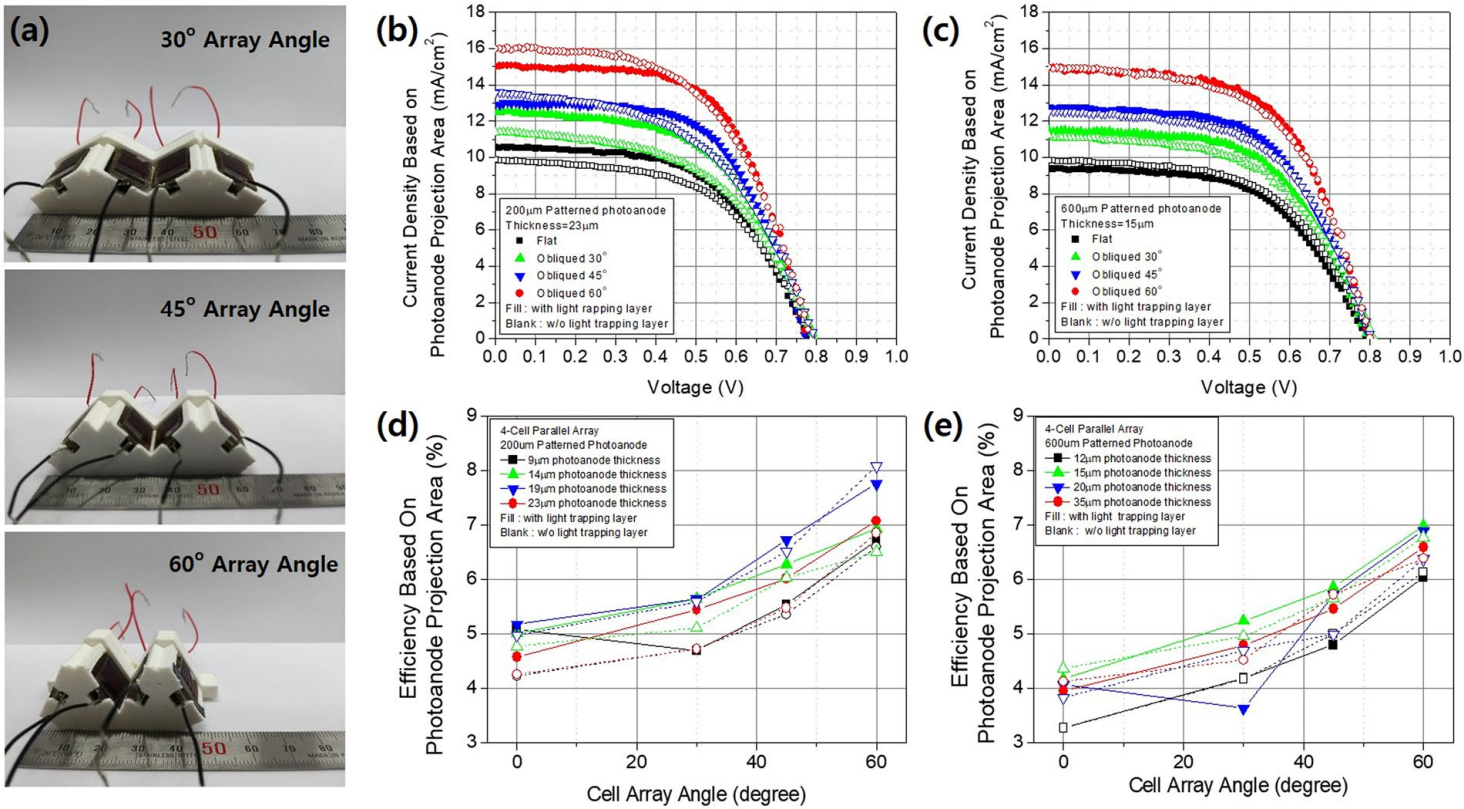
A photograph of (**a**) obliquely arrayed four-cell submodules at 30°, 45°, and 60° angles. The relationship between current density based on the photoanode projection area and voltage of (**b**) 200 µm patterned photoanodes and (**c**) 600 µm patterned photoanodes was affected by the presence of a light-trapping layer at all array angles tested. The efficiencies based on the photoanode projection areas of the (**d**) 200 µm patterned photoanodes and (**e**) 600 µm patterned photoanodes depended on the thickness and extent of light-trapping at all array angles tested. All measurements were made under a vertical incident light strength of 1 sun AM1.5.

The use of a light-trapping layer and a patterned photoanode mimicking leaf structures doubled DSSC conversion efficiency (from 4% to 8%) by modifying light distribution within cells and improving charge collection efficiency, shown at the modular scale in Fig. 5. The cells exhibited markedly improved efficiency when they were obliquely illuminated (Fig. 4). Therefore, if all DSSCs receive oblique light, total efficiency improves, reflecting the phyllotactic arrangement of leaves in the plant crown. To ensure such conditions, groups of four DSSCs were connected in parallel at defined array angles (Fig. 5a). Compared to the conventional flat array of most solar cells, the conversion efficiency of incident light increased with the array angle (Fig. 5b–e). Also internal resistance was decreased utilizing light trapping layer by increased angle of incident light by enhancing light travel length within patterned photoanode. Submodule conversion efficiencies varied by pattern size, light-trapping layer status, and photoanode thickness; in general, efficiency increased as the oblique angle of incident light rose. For example, the efficiencies of submodules 200 µm in pattern size with 23 µm thick photoanodes increased greatly in the presence of light-trapping layers, but the efficiencies of those with a 600 µm pattern size and 12 µm thick photoanodes did not.

However, it is clear that photoanode projection, area-based, submodule conversion efficiency was greatly improved (almost doubled) using crude imitations of leaf structures. We obtained a conversion efficiency of more than 8% even in the absence of a scattering layer, utilizing a semi-solid electrolyte and simple print fabrication. Further improvements will follow when the design, materials, and structure are optimized, creating next-generation solar cells.

**Figure 6:**
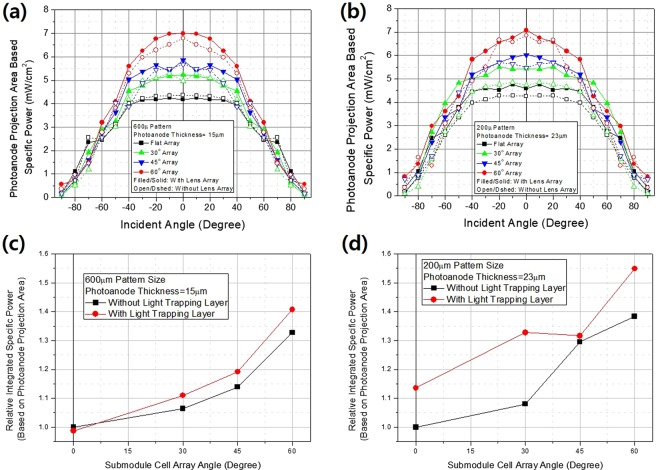
Power outputs by photoanode projection area and submodule incident angle of four-cell-arrayed submodules (different in terms of array angle) (**a**) for 600 µm patterned photoanodes (15 µm in thickness) and (**b**) 200 µm patterned photoanodes (23 µm in thickness) with and without light-trapping layers. Also shown are relative integrated specific powers at all incident angles afforded by the same four-cell-arrayed submodules for (**c**) 600 µm patterned photoanodes (15 µm in thickness) and (**d**) 200 µm patterned photoanodes (23 µm in thickness) with or without light-trapping layers. All measurements were performed under the 1 sun AM1.5 condition.

In terms of application to urban environments, we simulated electricity production over 1 day using a fixed installation (in terms of both place and angle) by changing the incident angle from 0 to vertical (Fig. 6). Both 200 µm patterned photoanodes 23 µm in thickness and 600 µm patterned photoanodes 15 µm in thickness exhibited large increases in electricity production. The light-trapping layer enhanced production in all cases, efficiently capturing the photons of omnidirectional incident light. The use of 200 µm patterned photoanodes 23 µm in thickness improved electricity production by almost 55% after introducing a light-trapping layer (compared to a conventional module design, thus a flat array without a light-trapping layer).

It is clear that our botanical approach is useful to improve PV cells for use in urban environments. We easily achieved a 55% enhancement of electricity production. The full potential of our method remains to be exploited. We have established new design rules for solar cells and modules, and we explored the underlying concepts and mechanisms. This work opens a new area of PV research.

## Conclusion

We have mimicked leaf arrangements and anatomy structure, taking a botanical approach toward the provision of tools for the creation of next-generation PVs for applications in urban environments. We create a light-trapping layer on top of the cells by mimic epidermis structure and we microscale-patterned the photoanodes by mimic palisade structure. We confirmed via 2D ray tracing that the light-trapping layer captured omnidirectional incident light and spread it homogeneously into the photoanodes. The light distribution and current densities were analyzed using an FEM. The light-trapping layer and photoanode patterning doubled DSSC conversion efficiency from 4% to 8% by modifying light distribution and improving charge collection efficiency. We expanded our work to the module scale by mimicking leaf arrangement further. DSSCs were much more efficient when they were obliquely illuminated. We connected groups of four DSSCs in parallel at appropriate array angles to greatly enhance module efficiency, just as the leaves in plant crowns exhibit a phyllotactic arrangement. Electricity production improved by almost 55% upon introducing a light-trapping layer (compared to that of a conventional modular design, thus a flat array lacking a light-trapping layer).

We established new design rules for solar cells and modules and explored the underlying concepts and mechanisms. We introduce a new field of PV cell research; our approach has great potential.

## Experimental details

### Calculations

The light distributions within photoanodes were calculated using 2D ray tracing methods implemented by an in-house code prepared in MATLab®. Each ray was refracted and reflected at the interface by reference to Snell’s Law and the Fresnel equations. The light intensities in photoanodes were calculated by averaging the energies passing through the photoanodes. The short circuit current density (Jsc) was obtained using the contiguity equations of the photoanodes, applied in a finite elements method (FEM) model using the Partial Differential Equation (PDE) Toolbox® of MATLab; light intensities were calculated by 2D ray tracing.

### Fabrication of light-trapping layers

Silicon wafers served as the substrates for master molding. For patterning on the mold, photoresist (PR, AZ 9260) was spun-coated (Midas System) onto wafers at 35,000 rpm for 30 s, yielding a coat 7 µm in thickness, and then the wafers were baked at 110 °C for 10 s. Photoresist was patterned photolithographically, followed by exposure to ultraviolet light using a mask aligner (MA-6, Suss Microtec); then the photoresist was removed by a developer (THMA 2.38% w/v) prior to engraving of 200 µm or 600 µm hexagonal arrays. We performed dry etching using a deep reactive ion etcher (SPTS); we cycled Bosch and isotropic etching. Then the etched Si wafers were cleaned. To fabricate light-trapping layers, polydimethysiloxane (PDMS SR-580, Heesung STS) coatings were applied using a master mold with a spin-coater (VSF-150MD) operating at 2,000 rpm for 40 s, and the wafers were baked at 70 °C for 20 min. Then the cured, PDMS light-trapping layer was peeled off the master mold. Etching was performed at the National Institute for Nanomaterials Technology, Pohang University of Science and Technology, Korea.

### DSSC preparation

F-doped SnO_2_ (FTO, sheet resistance 7Ω sq^−1^, Sigma-Aldrich) glass served as the electrode substrate. The glass was rinsed with acetone, ethanol, and deionized water under sonication for 30 min each time, and dried with nitrogen gas. The blocking layer (Solaronix) was deposited via automatic screen-printing (AutoMax) and heat-treated at 530 °C for 3 h in air. On the blocking layer, 20 nm TiO_2_ nanoparticles (Solaronix) were deposited over an area of 1 cm^2^, and the area was activated as were the uniform and patterned electrodes, followed by heating at 500 °C for 1 h in air. The TiO_2_-bearing FTO glass was immersed in a 0.3 solution (in ethanol) (Sigma-Aldrich) of N719 dye (Sigma-Aldrich) at room temperature for 20 h. To create counter-electrodes, Pt paste (Solaronix) was deposited onto FTO glass utilizing the doctor blade method (3M tape served as a mask) and then heated at 450 °C for 30 min in air.

A semi-solid electrolyte was deposited on both dye-loaded photoanodes and counterelectrodes utilizing the doctor blade method; 3M tape was used for masking. The semi-solid electrolyte was a mixture of 1.38 M 1-ethyl-3-methylimidazolium iodide (EMII, Sigma-Aldrich), 0.07 M iodine (Sigma- Aldrich), 0.13 M guanidine thiocyanate (Sigma-Aldrich), 0.85 M 4-tert-butylpyridine (Sigma-Aldrich), and 0.7 M lithium iodide (Sigma-Aldrich) in succinonitrile (SN, Sigma-Aldrich). The SN:EMII volume ratio was 3:1. And then Silica (SiO_2_) nanopowder (50nm, Amstec.) were added with weight ratio of SN to SiO_2_ nanopowder was 3.16:1. The two semi-solid electrodes were deposited on each photoanode and counter-electrode and then two electrodes were assembled using a press and sealed via ultraviolet curing (Laser Bond USA). Four DSSCs connected in parallel were arrayed on flat and oblique (30°, 45°, and 60°) frames to create sub-modules.

### Characterization

Field-emission scanning electron microscopy (FE-SEM, Hitachi S4800) was used to observe the surfaces and cross-sections of patterned electrodes and the light-trapping layers, and to measure electrode thicknesses. DSSC photovoltaic performance was evaluated using a solar simulator (Abet Technologies, model Sun 2000, 1,000 W Xenon source, Keithley 2400 source meter) under the 1 sun AM1.5 condition, and was calibrated using a KG-3 filter and an NREL-certified reference cell.

## Supplementary information

Supplementary Information
