# Supplementary material for: Leaf Anatomy and 3-D Structure Mimic to Solar Cells with light trapping and 3-D arrayed submodule for Enhanced Electricity Production
Source: Sci Rep. 2019 Jul 16;9:10273. doi: 10.1038/s41598-019-46748-x (PMC6635402; doi:10.1038/s41598-019-46748-x)
Supplement: Supplementary file 1 — Supplementary Information [file 41598_2019_46748_MOESM1_ESM.pdf]

Supplementary Information

Leaf Anatomy and 3-D Structure Mimic to Solar Cells with light trapping and 3-D arrayed submodule for Enhanced Electricity Production

Min Ju Yun[1], Yeon Hyang Sim[1,2], Seung I. Cha*[1,2], Dong Y. Lee[1,2]

1. Energy Conversion Research Center, Creative and Fundamental Research Division, Korea Electrotechnology Research Institute

2. Department of Electro-functionality Materials Engineering, University of Science and Technology

*Correspondence to Dr. Seung I. Cha, Korea Electrotechnology Research Institute, Boolmosan-ro 10beon-gil, Seongsan-gu, Changwon 51543, Korea. E-mail: sicha@keri.re.kr; Tel: +82-55-280-1649

Correspondence : sicha@keri.re.kr

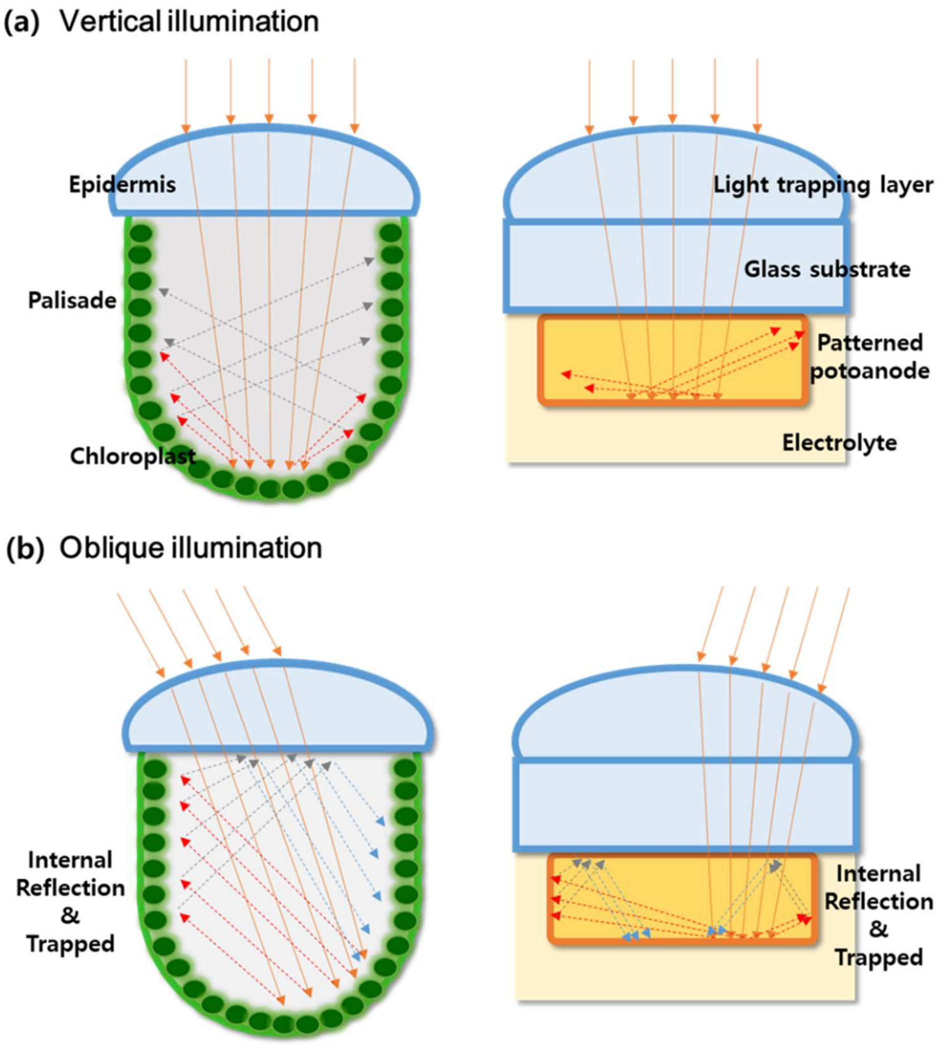

Figure S1. Schematic illustration of light trapping concept within patterned photoanode through light trapping layer at (a) vertical and (b) oblique incident light.

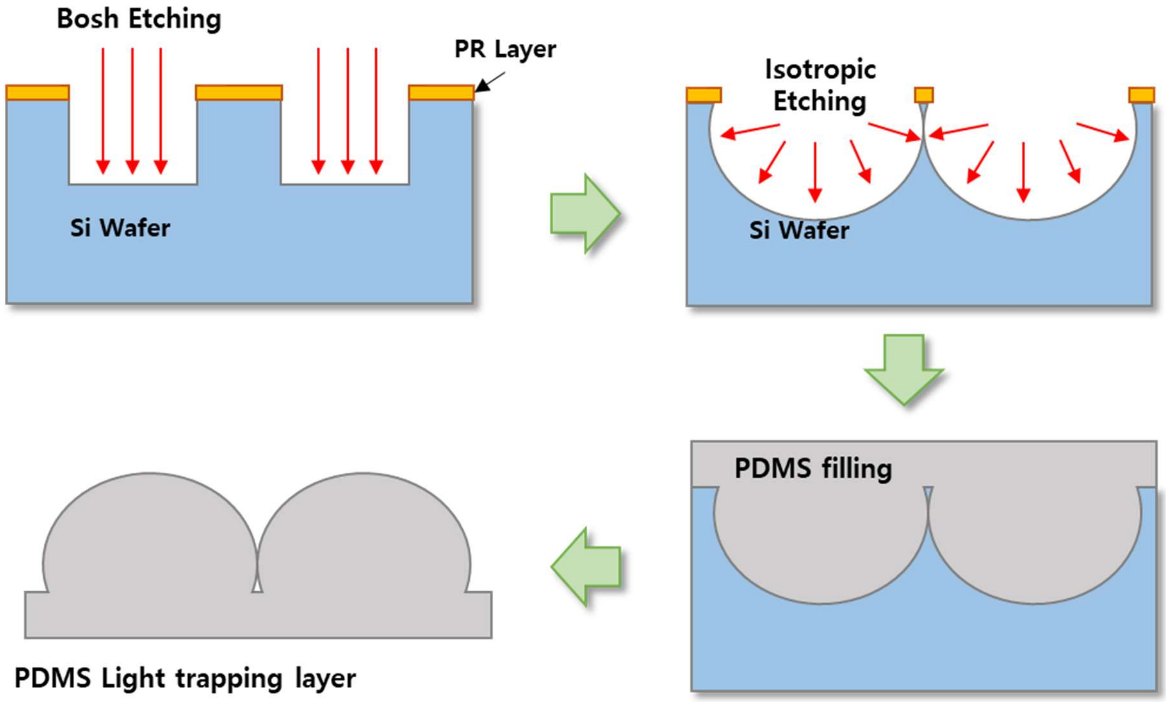

Figure S2. Schematic illustration of fabrication process of from Si wafer etching to molding light trapping layer.

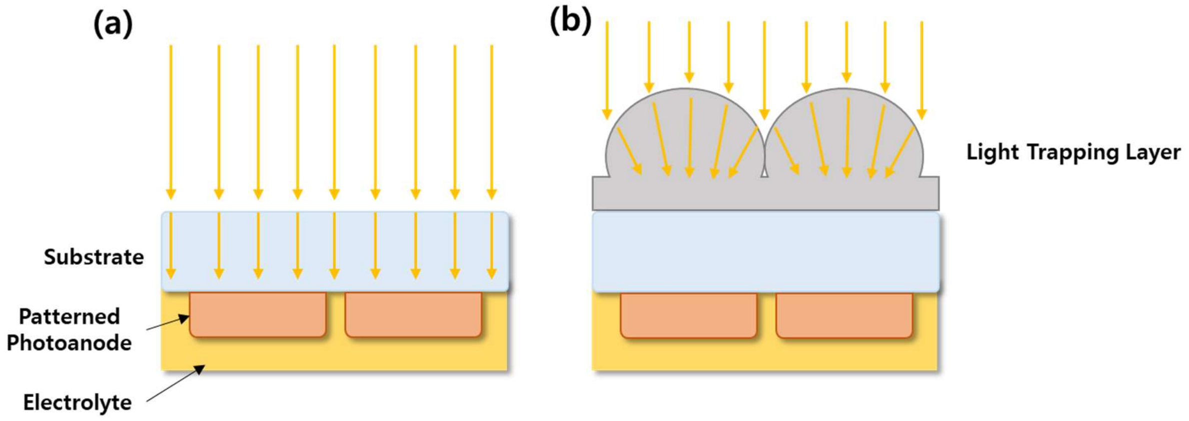

Figure S3. Schematic illustration of transmission spacing between patterns of photoanode of incident light (a) without light trapping layer and (b) with light trapping layer.

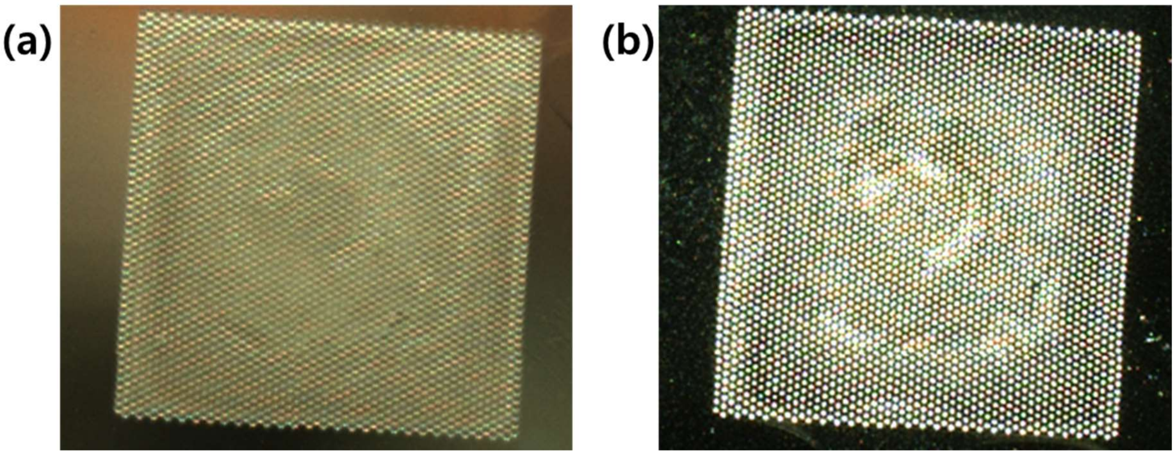

Figure S4. CCD images of (a) 200um sized light trapping layer and (b) light distribution and focusing through each patterns of light trapping layer by illumination LED source.

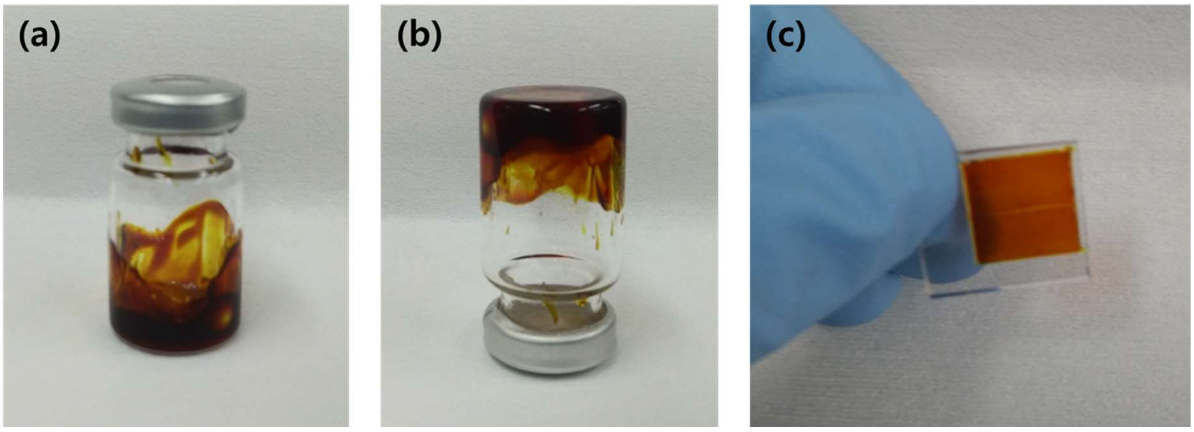

Figure S5. Photographs of (a) and (b) semi-solid electrolyte and (c) deposited semi-solid electrolyte on photoanode.

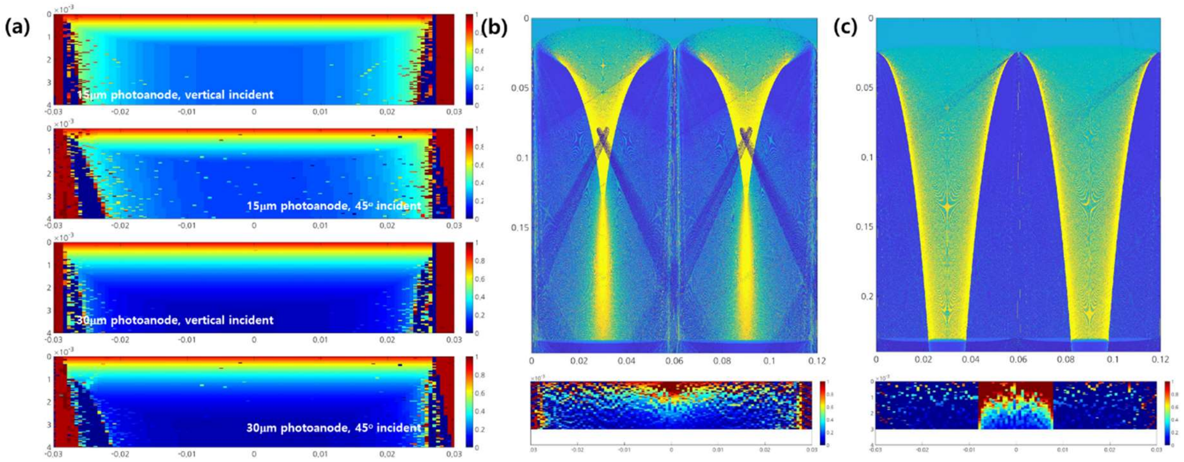

Figure S6. Light intensity distribution results within patterned photoanode (a) of 15 ㎛ and 30 ㎛ thickness without light trapping layer for vertical and 45 degree oblique incident light and (b) with light trapping layer (600 ㎛ sized pattern) of 10 ㎛ height and (c) 50 ㎛ height by 2D ray tracing analysis.
